# Fibroblast growth factor receptor alterations and resistance mechanisms in the treatment of pediatric solid tumors

**DOI:** 10.20517/cdr.2024.208

**Published:** 2025-06-06

**Authors:** Ivan Li, Yuchen Huo, Ting Yang, Howard Gunawan, Ludmil B. Alexandrov, Peter E. Zage

**Affiliations:** ^1^Tufts University, Medford, MA 02155, USA.; ^2^Department of Pediatrics, Division of Hematology-Oncology, University of California San Diego, La Jolla, CA 92093, USA.; ^3^Departments of Cellular and Molecular Medicine and Bioengineering, University of California San Diego, La Jolla, CA 92093, USA.; ^4^Peckham Center for Cancer and Blood Disorders, Rady Children's Hospital, San Diego, CA 92123, USA.; ^#^They contributed equally.

**Keywords:** FGFR, pediatric cancer, neuroblastoma, resistance, targeted therapy

## Abstract

**Aim:** The fibroblast growth factor receptor (FGFR) family receptors regulate cell proliferation, survival, and migration and are linked to cancer drug resistance. FGFR gene family alterations have been found in multiple adult cancers, for which FGFR inhibitors are in various stages of clinical development. This study aimed to delineate the FGFR alterations in pediatric tumors and provide a preclinical rationale for developing FGFR inhibitors for select pediatric patients.

**Methods:** The prevalence of FGFR alterations in pediatric cancers was calculated from databases with available pediatric tumor data. Effects of the pan-FGFR inhibitor infigratinib (BGJ398) on pediatric cancer cell line viability and migration were evaluated by continuous live cell imaging and compared to FGFR gene expression. Effects on cell death and signaling pathway activity were evaluated by live cell imaging and Western blots.

**Results:** Overall rates of FGFR1-4 gene alterations in pediatric cancers were rare, and the mutation profile substantially differs from that of adult tumors. Although FGFR genomic alterations are rare in pediatric neuroblastoma tumors, overexpression of FGFR1-4 is observed in tumor subsets and is associated with outcomes. Dose-dependent inhibition of cell proliferation and migration and promotion of cell death were achieved with BGJ398 treatment in neuroblastoma cell lines, accompanied by inhibition of RAS-MAPK pathway activity and induction of apoptosis.

**Conclusion:** Adult and pediatric cancers share common mechanisms of FGFR activation but differ in overall alteration rates and relative abundance of specific aberrations. Preliminary experimental data indicate the therapeutic potential of FGFR inhibitors and suggest mechanisms of resistance in the treatment of pediatric cancers.

## INTRODUCTION

Cancer is one of the leading causes of death in children and adolescents and approximately 300,000 children aged 0 to 19 years old are diagnosed with cancer globally each year^[1]^. Pediatric brain tumors and other extracranial solid tumors such as neuroblastoma, rhabdomyosarcoma, and Ewing sarcoma are common forms of cancer in children^[2]^. Currently, the causes of these cancers are mostly unknown, and the pathways and signals involved in the growth and spread of pediatric cancer cells and in their responses to therapy are not well understood. Furthermore, children with aggressive solid tumors frequently develop treatment resistance and have extremely poor chances of cure despite intensive therapies, and the rare survivors experience significant side effects and complications, including long-term developmental risks, as a consequence of chemo- and radiation therapies^[3,4]^. Children with these tumors urgently need new treatment options. Targeted therapies designed to address specific cancer pathways critical for tumor survival and treatment resistance have the potential to improve survival and cure rates while reducing the frequency and severity of long-term side effects. However, progress in developing targeted therapies for pediatric cancers has significantly lagged behind advancements made for adults.

Abnormal expression and activity of many growth factors and/or their receptors contribute to the initiation and growth of many cancer types, which has been more extensively studied in adult cancers. Epidemiological and molecular studies have reported a variety of genetic alterations in cancer cells, including mutations, fusions, gene amplifications, and gene overexpression. Many of these changes lead to constitutive activation of receptors and downstream signaling pathways, resulting in uncontrolled cell survival, proliferation, and migration, which are hallmarks of cancer. In the past 20 years, a number of successful targeted cancer therapies have been developed to address cancers driven by growth factor receptor oncogenic activation.

The fibroblast growth factor receptor (FGFR) family consists of four members, FGFR1-4, that can regulate multiple physiological processes such as endocrine homeostasis, wound repair, and cellular behaviors (including proliferation, differentiation, migration, and survival), via activation of fibroblast growth factor receptor substrate 2 (FRS2), mitogen-activated protein kinase (MAPK)/extracellular signal-regulated kinase 1/2 (ERK1/2), phosphoinositide 3-kinase (PI3K)/protein kinase B (AKT) signaling pathways, signal transducer and activator of transcription 3 (STAT3), phospholipase Cγ (PLCγ), and ribosomal protein S6 kinase 2 (RSK2). Given the role of FGFs and FGFRs in cell and tissue development and function, they have been rapidly linked to tumorigenesis and chemoresistance occurring during anti-cancer therapy. Indeed, these FGFR family genes have been linked to treatment resistance in a variety of adult cancers^[5]^.

Common FGFR genomic alterations include gene amplifications, gene mutations or single nucleotide variants (SNVs), and structural variants (SVs), which promote stemness, proliferation, invasion, and drug resistance in cancer cells. Genetic alterations, including fusions, mutations, and amplification or overexpression of the FGFR genes, have been found in multiple types of adult cancers, such as intrahepatic cholangiocarcinoma, urothelial cancer, and gastric cancer^[6]^. In a recent study, activating FGFR alterations were found in approximately 3% (41/1,395) of pediatric solid tumors^[7]^. Prior studies have also shown that pediatric cancers such as rhabdomyosarcoma (RMS) harbor recurrent *FGFR4* sequence variants and low-grade gliomas (LGG) harbor recurrent *FGFR1*/*2* variants and fusions, including intragenic duplications in *FGFR1* affecting the tyrosine kinase domain^[8-10]^. These alterations typically lead to constitutive receptor activity and activation of downstream pathways, resulting in tumor initiation and progression^[11,12]^ via inhibition of apoptotic signaling as well as induction of angiogenesis and epithelial-mesenchymal transition (EMT)^[5]^.

Small molecule FGFR inhibitors such as pemigatinib, infigratinib (BGJ398), and futibatinib have been shown to be effective in the clinic in selected adult cancer patients^[13]^. However, with the exception of a few reports^[14-18]^, the efficacy of these and other FGFR inhibitors against pediatric solid tumors has not been well established. In this report, we first queried cancer databases to delineate the presence and prevalence of FGFR gene family alterations in pediatric cancers across various tumor types. We then experimentally tested a selective pan-FGFR inhibitor, BGJ398, in selected pediatric cancer cell lines with the goal of providing a preclinical rationale for developing FGFR inhibitors for select pediatric cancer patients.

## METHODS

### Therapeutic agents

BGJ398 was generously provided by QED Therapeutics (San Francisco, CA). A 10mM stock solution was generated in 100% DMSO (Sigma-Aldrich) and stored at -20 °C. BGJ398 was diluted in phosphate-buffered saline (PBS) immediately before use.

### Database queries

To determine the prevalence of FGFR gene family alterations across various pediatric cancer types, we performed systematic queries and extraction of relevant information from clinical databases that house histological and molecular profiling data of pediatric cancers, including brain tumors, sarcomas, and neuroblastoma. We define pediatric cancer patients as those whose initial diagnosis of cancer took place between the ages of 0 and 18 years.

In cBioPortal databases (http://www.cbioportal.org), pediatric cancer studies for individual cancer types (brain tumors, sarcomas, and neuroblastoma) were chosen, followed by “Query by Gene”, with both “Mutation” and “Copy number alterations” selected. Query results were graphed and tabulated with “OncoPrint”. Additionally, FGFR1/2/3/4 gene alterations were queried for their mutual exclusivity from other oncogenic mutations in *IDH1*, *IDH2*, *KRAS*, *ALK*, *ROS1*, *NTRK1*, *NTRK2*, *NTRK3*, *BRAF*, and *EGFR*. For more detailed analysis, under “Query by Gene”, both “Mutation” and “Copy number alterations” were selected for “*FGFR1*, *FGFR2*, *FGFR3*, *FGFR4*”. In the Foundation Medicine database (https://corpsite.foundationmedicine.com/insights-and-trials), the analysis was focused on “ALTERATIONS” in “brain glioma” in patients aged 0-18 years.

We obtained microarray analysis results of 649 individual neuroblastoma patient tumor samples, 85 Ewing sarcoma patient tumor samples, and 101 rhabdomyosarcoma patient tumor samples from the R2 Genomics Analysis and Visualization Platform (http://r2.amc.nl) using the Kocak, Dirksen, and Williamson databases, respectively^[19-21]^. *FGFR1*, *FGFR2*, *FGFR3*, and *FGFR4* probesets in each database with the highest average signals were selected for analysis. Kaplan-Meier analyses were performed online and the resulting survival curves and p-values (obtained via the Log-Rank test) were downloaded as previously described^[22]^.

### Cell proliferation, migration, and apoptosis

A panel of established pediatric cancer cell lines^[14,22-24]^ was used to screen for sensitivity to FGFR inhibition. Cell viability with and without treatment with the FGFR inhibitor BGJ398 was analyzed using modified methyl thiazolyl tetrazolium (MTT, Sigma-Aldrich; St. Louis, MO) assays and continuous live cell imaging as previously described^[25,26]^. Briefly, cells were plated in 96-well plates and either left untreated or were treated with increasing concentrations of BGJ398. Percent cell viability was calculated by subtracting the optical density (OD) of media alone as background and then dividing by the OD from control, untreated cells. For live cell imaging assays, 96-well plates treated as above with BGJ398 were placed into the IncuCyte® Zoom^TM^ system (Essen Bioscience, Ann Arbor, MI) and percent cell confluence was calculated from phase contrast images taken every 6 h using IncuCyte® analysis software. For cell viability and confluence assays, replicates of at least three wells were used for each experimental condition, and assays were performed with at least three biologically independent replicates. Cell viability and confluence time- and dose-response curves were generated from calculated values, and IC50 values were derived as published^[25,26]^.

Cell migration was evaluated using the IncuCyte Zoom^TM^ 96-Well Scratch Wound Cell Migration assay as previously published^[14,27]^. Cells were seeded in 96-well ImageLock tissue culture plates (Essen BioScience) with 12 replicates per experiment. Plates were incubated overnight to allow for cell adhesion, and then identical individual scratches were made in each well using the Wound Maker tool (Essen BioScience). Wells were gently washed twice with culture media to remove dislodged cells, and plates were placed in the IncuCyte Zoom^TM^. Phase contrast images were taken every 3 h for 48 h using a 10X objective. Wound width was calculated using IncuCyte Zoom^TM^ software, and treated and untreated cells were compared quantitatively for inhibition of migration.

To evaluate the effects of BGJ398 on cell death, cells were plated and monitored via live cell imaging as above^[26]^. Cells were treated with increasing concentrations of BGJ398, and 5 μM IncuCyte® Caspase 3/7 Green Reagent (Essen Bioscience) was added to individual wells. Four non-overlapping phase contrast and fluorescent images were taken of each well every 6 h for 72 h in total, as described above. Green objects representing individual cells with activated caspases were counted using IncuCyte Zoom^TM^ software, and average counts per field were generated and normalized to control. Replicates of at least three wells were used for each experiment, and assays were performed at least three independent times as above.

### Signaling pathway analysis

Intracellular signaling pathways that may be involved in tumor cell responses to FGFR inhibition were analyzed by Western blotting as described^[26]^. Briefly, cells were plated in 6-well plates or 10 cm tissue culture dishes and allowed to adhere overnight. Cells were then treated with either BGJ398 or vehicle for designated times using specified doses. Treated cells were harvested using non-enzymatic approaches and lysed using RIPA buffer supplemented with Pierce Protease inhibitor and phosphatase inhibitor (Life Technologies).

Protein concentrations in samples were measured using BCA Protein Assay Kits (Thermo Fisher Scientific, San Diego, CA), and equal amounts of protein were loaded in individual lanes of 4%-12% Bis-Tris gels (Invitrogen, Carlsbad, CA) containing 3-(*N*-morpholino)propanesulfonic acid (MOPS) sodium dodecyl sulfate (SDS) running buffer (Life Technologies). Proteins separated in gels were transferred to polyvinylidene difluoride (PVDF) membranes (Thermo Fisher) using the iBlot2 Dry Blotting transfer system (Invitrogen) or using the Invitrogen Mini Blot Module and Novex Transfer Buffer (Life Technologies).

Membranes were blocked in 5% BSA in TBS with 0.1% Tween 20 for one hour at room temperature and then incubated overnight at room temperature with primary antibodies (all from Cell Signaling, Danvers, MA, except as noted below; all antibodies were diluted in TBST blocking buffer to achieve specific concentrations) to total MEK1/2 (9126), phosphorylated MEK1/2 (9154), total ERK1/2 (4695), phosphorylated ERK1/2 (4370), poly-ADP ribose polymerase (PARP) (9542), Glyceraldehyde-3-phosphate dehydrogenase (GAPDH) (5174S), total FGFR1 (9740;), phosphorylated FGFR1 (2544), total FRS-2 [sc-17841; Santa Cruz Biotechnology (Dallas, TX)], and phosphorylated FRS2 (Try196; 3864S).

Membranes were then washed 3 times with PBS with 0.1% Tween-20 and incubated for one hour at room temperature with secondary HRP-conjugated anti-rabbit (W401B; 1:5000; Promega, Madison, WI) or anti-mouse (W402B; 1:5000; Promega) IgG antibodies. Protein bands on the membranes were exposed using Amersham ECL Prime Luminol Enhancer and Peroxide Solution (GE Healthcare, Piscataway, NJ), followed by SuperSignal™ West Pico Plus Chemiluminescent Substrate (Thermo Fisher). Membranes were exposed to Denville Scientific HyBlot CL Films (Thomas Scientific, Swedesboro, NJ), and individual films were developed in an ECOMAX™ x-ray film processor (Protec, Oberstenfeld, Germany).

### RNA-seq data analysis

Raw FASTQ files were first trimmed by Trim Galore (v0.6.10)^[28]^, followed by ribosomal RNA removal using SortMeRNA (v4.3.6)^[29]^. STAR (v2.7.10b)^[30]^ was applied to align FASTQ files to the human genome build GRCh38.d1.vd1 and generate BAM files. StringTie (v2.2.1)^[31]^ was applied to assemble and quantify transcripts, while FeatureCounts (v2.0.6)^[32]^ was used to count and summarize reads.

### Statistical Analysis

The relationships between FGFR1-4 gene expression and IC50 values were evaluated using Spearman correlation coefficients and corresponding p-values. Pearson correlation coefficients were also calculated to provide additional insight into the linear associations between FGFR gene family expression and BGJ398 IC50 values. Correlation lines were denoted as red when Pearson correlation *P* values were < 0.05.

## RESULTS

### Prevalence of FGFR gene alterations in pediatric cancers

**Figure 1:**
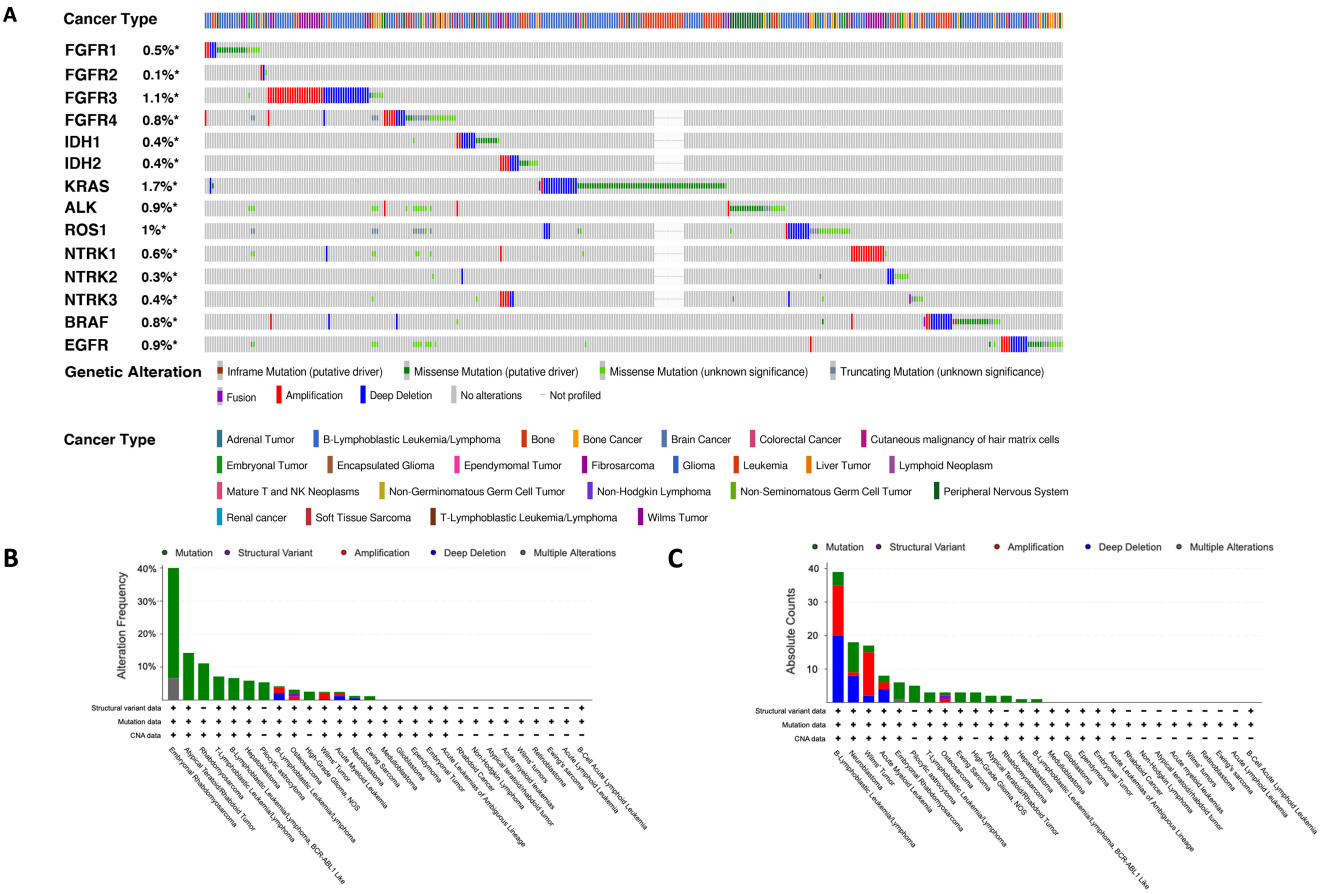
Prevalence of targetable FGFR signaling pathway alterations in pediatric cancers. Queries and resulting figures were generated in cBioPortal (http://www.cbioportal.org) by searching Pediatric Cancer Studies and querying by FGFR1, FGFR2, FGFR3, FGFR4, IDH1, IDH2, KRAS, ALK, ROS1, NTRK1, NTRK2, NTRK3, BRAF, and EGFR, with the resulting OncoPrint for identified mutations, amplifications, and deletions shown (A). (B) FGFR1, FGFR2, FGFR3, and FGFR4 gene alteration frequency (B) and absolute counts (C) by pediatric cancer type from cBioPortal are shown. FGFR: fibroblast growth factor receptor.

Queries of pediatric cancers with FGFR alterations were generated using cBioPortal. Using a broad search of all pediatric cancers, we observed the rates of alterations of the *FGFR1*, *FGFR2*, *FGFR3*, and *FGFR4* genes in pediatric cancers to be 0.5%, 0.1%, 1.1%, and 0.8%, respectively. Results also show that FGFR gene family alterations in the vast majority of cases are mutually exclusive from each other and from other known oncogenic alterations [Figure 1A]. By frequency, pediatric soft tissue sarcoma had the highest percentage of FGFR1-4 gene alterations, followed by atypical teratoid-rhabdoid tumors (ATRT), pediatric T-cell acute lymphoblastic leukemia (T-ALL), and B-cell lymphoblastic ALL (B-ALL), [Figure 1B and C].

### FGFR gene alterations in pediatric brain tumors, sarcomas, and neuroblastoma

**Figure 2:**
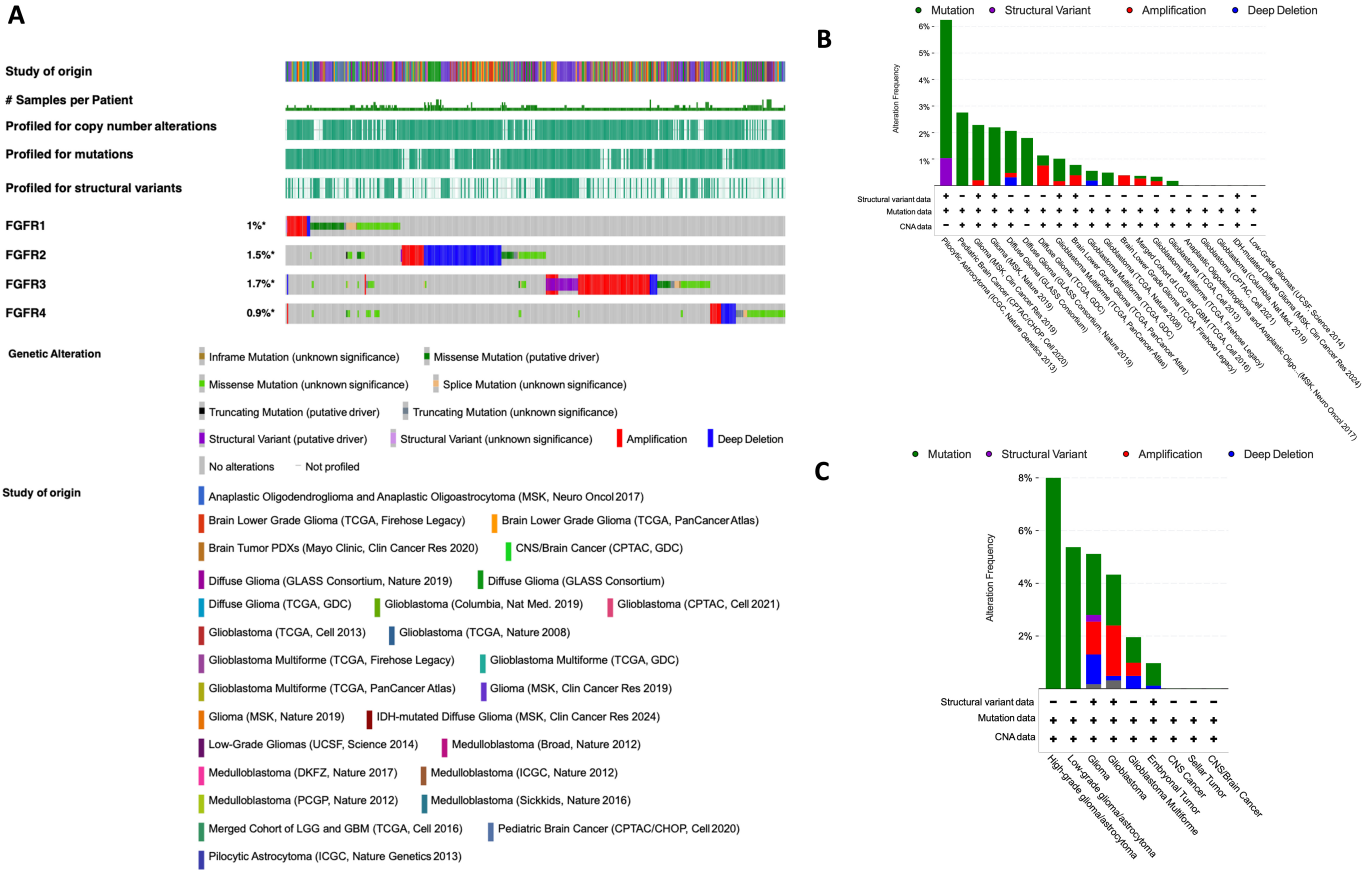
Types and prevalence of FGFR alterations in brain tumors. Queries and resulting figures were generated in cBioPortal (http://www.cbioportal.org) by searching for “brain” and querying by FGFR1, FGFR2, FGFR3, FGFR4, IDH1, IDH2, KRAS, ALK, ROS1, NTRK1, NTRK2, NTRK3, BRAF, and EGFR. (A) OncoPrint display of identified mutations, amplifications, and deletions; (B) FGFR1-4 gene alteration frequency by individual study; (C) Frequency of FGFR1-4 alteration by brain cancer type. FGFR: fibroblast growth factor receptor.

**Figure 3:**
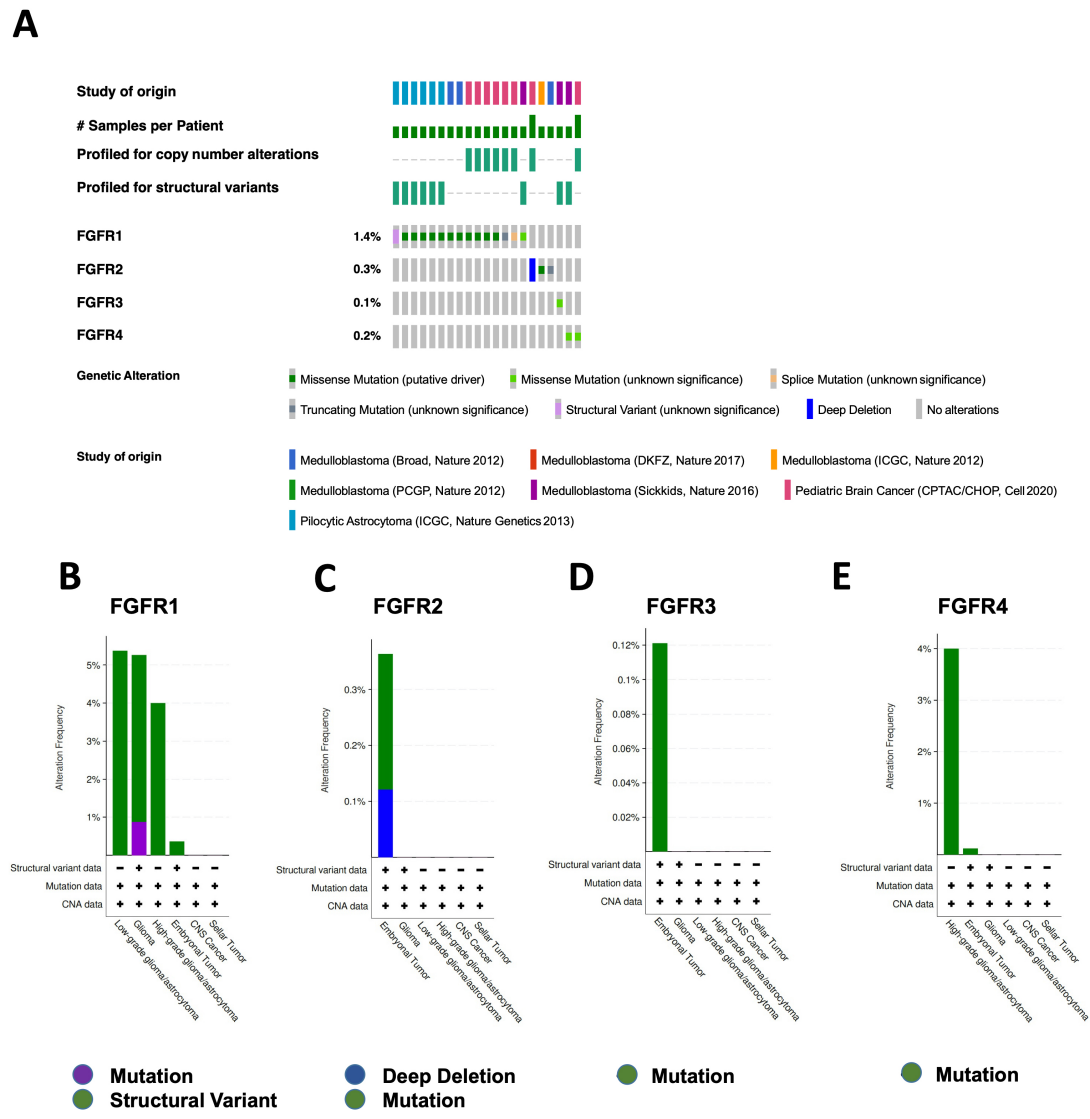
Alteration frequencies of FGFR genes in pediatric brain tumors. Queries and resulting figures were generated in cBioPortal (http://www.cbioportal.org) by searching “brain” and selecting all pediatric cancer studies and querying by FGFR1, FGFR2, FGFR3, and FGFR4. (A) OncoPrint display of identified mutations, amplifications, and deletions. (B) FGFR1, (C) FGFR2, (D) FGFR3, and (E) FGFR4 gene alteration frequency by pediatric brain tumor type. FGFR: fibroblast growth factor receptor.

The rates of alterations of the *FGFR1*, *FGFR2*, *FGFR3*, and *FGFR4* genes in brain tumor patient samples were 1%, 1.5%, 1.7%, and 0.9%, respectively [Figure 2A], and pediatric brain tumors had among the highest levels of FGFR gene family alterations among all brain tumor databases [Figure 2B]. Among all brain tumors, gliomas had the highest rates of FGFR gene alterations [Figure 2C]. In combined pediatric brain tumor databases, the rates of alterations of the *FGFR1*, *FGFR2*, *FGFR3*, and *FGFR4* genes were 1.4%, 0.3%, 0.1%, and 0.2%, respectively, with *FGFR1* and *FGFR4* gene alterations found in up to 5% of pediatric gliomas while *FGFR2* and *FGFR3* alterations were less common but found most frequently in medulloblastoma [Figure 3]. FGFR missense gene mutations were the predominant type of alterations in pediatric gliomas. Frequent alterations include *FGFR1* N546K, *FGFR1* K656E, *FGFR3* K650E, and FGFR1-3 fusions. Furthermore, from the alteration frequency histograms, mutations are more frequent in the *FGFR1* and *FGFR4* genes, deep deletions are more frequent in the *FGFR2* gene, and *FGFR3* gene amplifications are common [Figures 2 and 3]. Although no *FGFR3* gene fusions were found in pediatric gliomas, *FGFR1* K656E mutation is more frequent and appears to be the dominant form of mutation. Comparing adult and pediatric GBM tumor profiles, *FGFR3* gene alterations are only found in cases of adult GBM, including *FGFR3*-*TACC3* gene fusions (2.4%) and *FGFR3* K650E mutations (0.2%), while *FGFR1* gene alterations, specifically *FGFR1* K656E mutations, are ~ 7 times more frequent (2.2%) in pediatric glioma tumors compared to adult gliomas. Overall, the *FGFR* gene alteration rate is lower in pediatric than in adult gliomas [Table 1].

**Table 1:** FGFR alteration profiles in pediatric *vs.* adult glioblastoma patient tumors

	**FGFR3-TACC3 Prevalence (%)**	**FGFR3 K650E Prevalence (%)**	**FGFR1 K656E Prevalence (%)**
All age	2.3	0.2	0.3
Pediatric	0	0	2.2
Adult	2.4	0.2	0.3

FGFR: fibroblast growth factor receptor.

**Figure 4:**
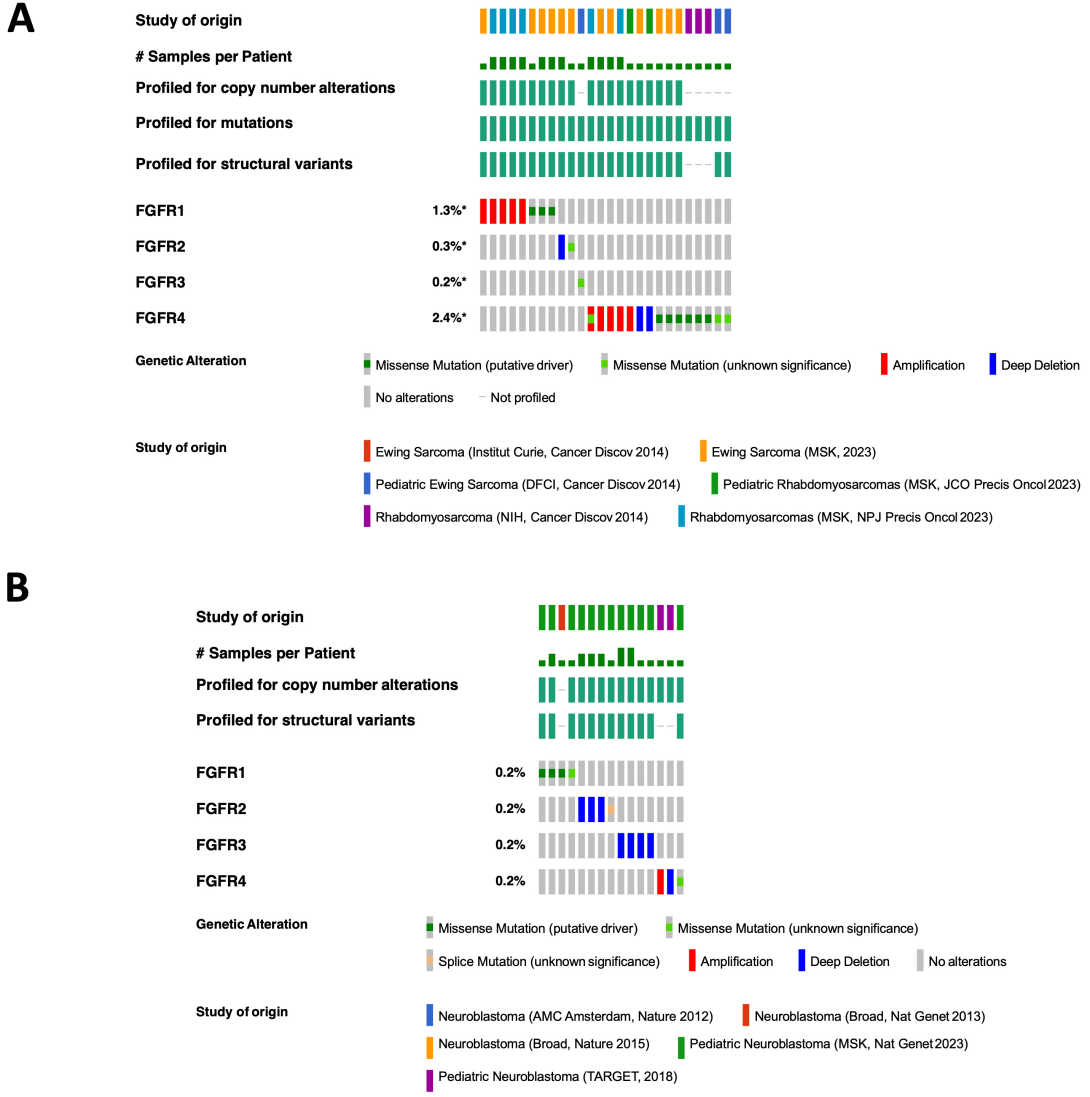
Types of FGFR alterations in pediatric sarcomas and neuroblastoma. Queries and resulting figures were generated in cBioPortal (http://www.cbioportal.org) by searching “sarcoma” and “neuroblastoma” separately and selecting all pediatric cancer studies and querying by FGFR1, FGFR2, FGFR3, and FGFR4, with the resulting OncoPrint for identified mutations, amplifications, and deletions shown for pediatric sarcomas^[41,89-92]^ (A); and neuroblastoma^[93-95]^ (B). FGFR: fibroblast growth factor receptor.

**Figure 5:**
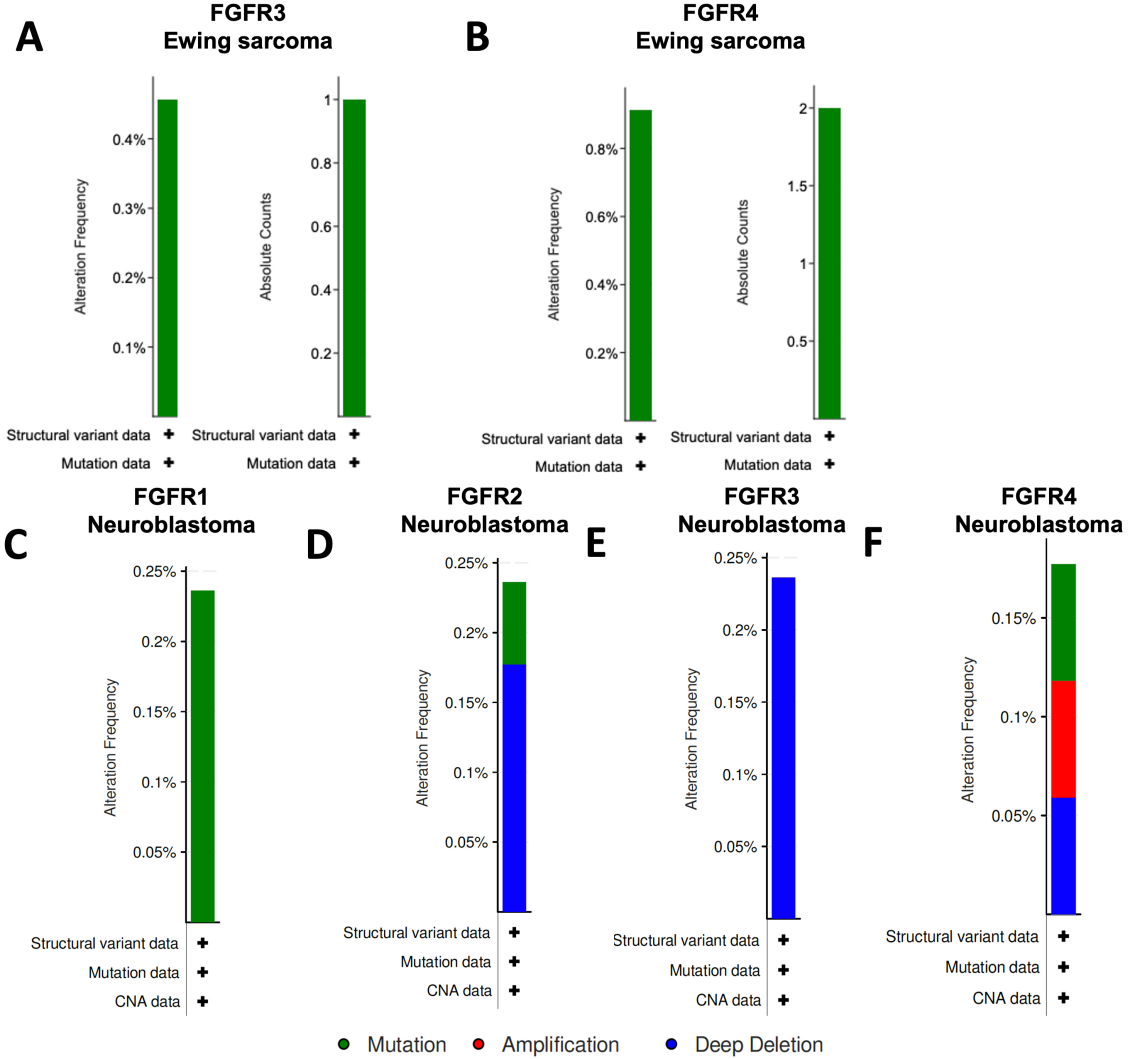
Alteration frequencies of FGFR genes in Ewing sarcoma and neuroblastoma. Queries and resulting figures were generated in cBioPortal (http://www.cbioportal.org) by searching “sarcoma” and “neuroblastoma” and selecting all pediatric cancer studies and querying by FGFR1, FGFR2, FGFR3, and FGFR4, with (A) FGFR3 and (B) FGFR4 gene alteration frequencies and absolute counts for Ewing sarcoma are shown. No mutations or other gene variations were found for the FGFR1 and FGFR2 genes in Ewing sarcoma samples. (C) FGFR1, (D) FGFR2, (E) FGFR3, and (F) FGFR4 gene alteration frequencies for neuroblastoma tumors from cBioPortal are shown. FGFR: fibroblast growth factor receptor.

**Figure 6:**
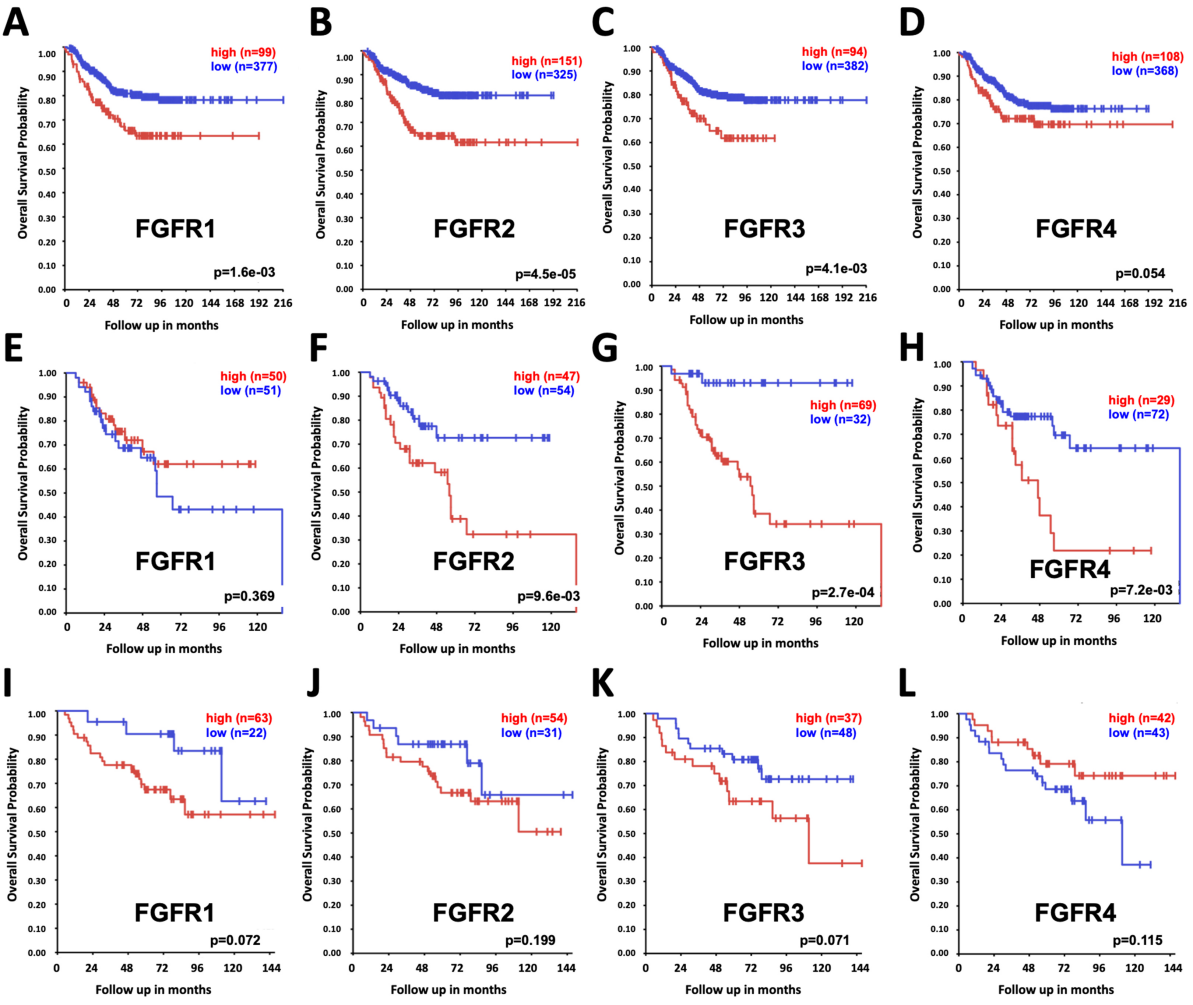
Association of pediatric patient outcomes with FGFR gene family expression levels. Using the Kocak dataset from the R2 Genomics Analysis and Visualization Platform^[19]^ (http://r2.amc.nl; accessed on 10/15/2024), Kaplan-Meier curves were generated and downloaded depicting overall survival (OS) among all neuroblastoma patients, separated by tumors with high expression (in red) *vs.* low expression (in blue) of FGFR1 (A); FGFR2 (B); FGFR3 (C); and FGFR4 (D), with patient numbers in parentheses. Using the Williamson and Dirksen datasets^[20,21]^ from the R2 Genomics Analysis and Visualization Platform (http://r2.amc.nl; accessed on 10/16/2024), Kaplan-Meier curves were generated and downloaded depicting overall survival (OS) among all with rhabdomyosarcoma (E-H) and Ewing sarcoma (I-L), separated by tumors with high expression (in red) *vs.* low expression (in blue) of FGFR1 (E,I), FGFR2 (F,J), FGFR3 (G,K), and FGFR4 (H,L), with patient numbers in parentheses. FGFR: fibroblast growth factor receptor.

In pediatric sarcoma databases, the rates of alterations of the *FGFR1*, *FGFR2*, *FGFR3*, and *FGFR4* genes were 1.3%, 0.2%, 0.2%, and 2.4%, respectively, while in neuroblastoma tumor samples, the rates of alterations were 0.2% for each [Figure 4]. In Ewing sarcoma tumor samples, no alterations of either *FGFR1* or *FGFR2* were identified, while *FGFR3* and *FGFR4* mutations were identified in less than 1% of samples [Figure 5A and B]. In neuroblastoma patient tumor samples, amplifications, deletions, and mutations for FGFR gene family members were identified in small percentages of tumors [Figure 5C-F]. Despite the limited numbers of tumors with FGFR gene family alterations, however, high expression of the *FGFR1*, *FGFR2*, and *FGFR3* genes in tumor samples were each associated with worse outcomes for children with neuroblastoma, with a trend toward worse outcomes in patients with high *FGFR4* tumor expression as well [Figure 6A-D]. Gene expression profiles of neuroblastoma patient tumors identified subsets of neuroblastoma tumors with FGFR1-4 overexpression, upwards of two standard deviations (z-score > 2) from the mean relative FGFR expression levels [Supplementary Figure 1], suggesting that subsets of neuroblastoma tumors are likely more sensitive to FGFR inhibition despite the relatively small percentage of tumors with gene alterations. By comparison, elevated *FGFR2*, *FGFR3*, and *FGFR4* gene expression levels demonstrated associations with worse outcomes for children with rhabdomyosarcoma tumors, but no significant associations with FGFR gene family member expression were identified for children with Ewing sarcoma [Figure 6E-L].

### Efficacy of FGFR inhibition in pediatric tumor cell lines

**Figure 7:**
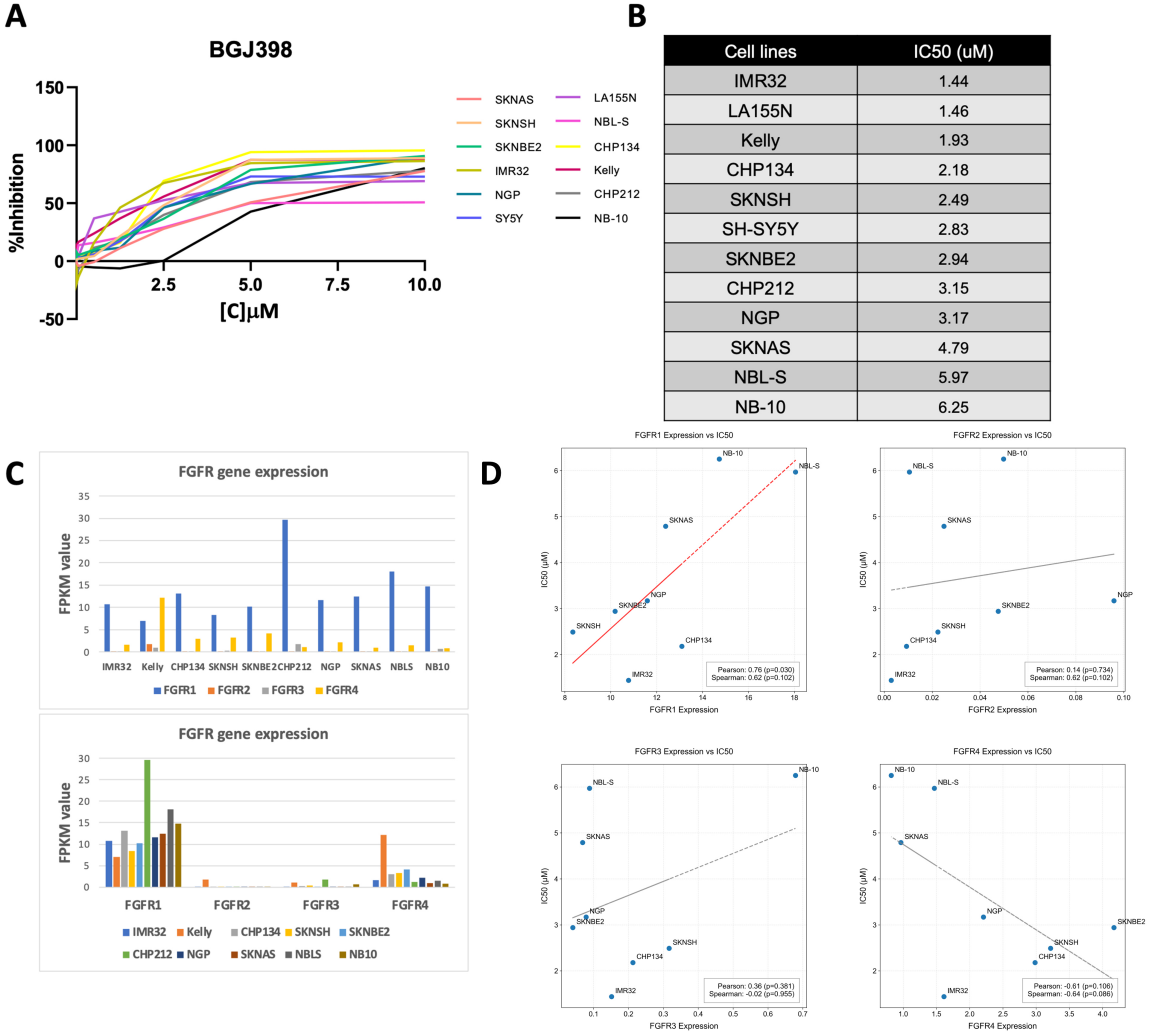
Effect of FGFR inhibitor BGJ398 on neuroblastoma cell proliferation and survival. (A) Neuroblastoma cell lines (SK-N-AS, SK-N-SH, SK-N-BE(2), IMR-32, NGP, SH-SY5Y, LA155N, NBL-S, CHP134, Kelly, CHP212, and NB-10) were treated with increasing concentrations of BGJ398 for 72 h. Mean average values for a reduction in confluence (% inhibition) from triplicate experiments were then plotted in Microsoft Excel against BGJ398 dose levels; (B) IC50 values were calculated using curve-fit equations for each tested neuroblastoma cell line listed from lowest to highest IC50 values for each cell type; (C) Relative FGFR1-4 gene expression levels in cell lines were obtained by RNA-sequencing and were graphed in Microsoft Excel as fragments per kb of transcript per million fragments mapped (FPKM); and (D) FGFR gene member expression was compared to calculated IC50 values using Python Code software. FGFR: fibroblast growth factor receptor; BGJ398: infigratinib.

To evaluate the potential efficacy of FGFR inhibition in pediatric tumor cell lines, we evaluated the pan-FGFR inhibitor BGJ398 for effects on cell proliferation, migration, and intracellular signaling. In neuroblastoma cell lines, BGJ398 treatment resulted in over 50% reduction in cell confluence and viability in nearly all tested cell lines [Figure 7A and B; Supplementary Figures 2 and 3]. Of 12 neuroblastoma cell lines tested, IMR32 was the most sensitive to BGJ398 (IC50 = 1.44 μM) whereas SJ-NB-10 was the most resistant (IC50 = 6.25 μM) [Figure 7A and B]. Responses to BGJ398 also correlated with *FGFR4* gene expression, with higher *FGFR4* expression leading to increased sensitivity, but inversely to *FGFR1* gene expression, with higher *FGFR1* expression leading to increased resistance and higher IC50 values [Figure 7C and D], suggesting FGFR gene expression could serve as a biomarker for potential response and resistance. BGJ398 treatment also resulted in reduced viability of all tested pediatric Ewing sarcoma and rhabdomyosarcoma cell lines at similar doses [Supplementary Figure 4].

**Figure 8:**
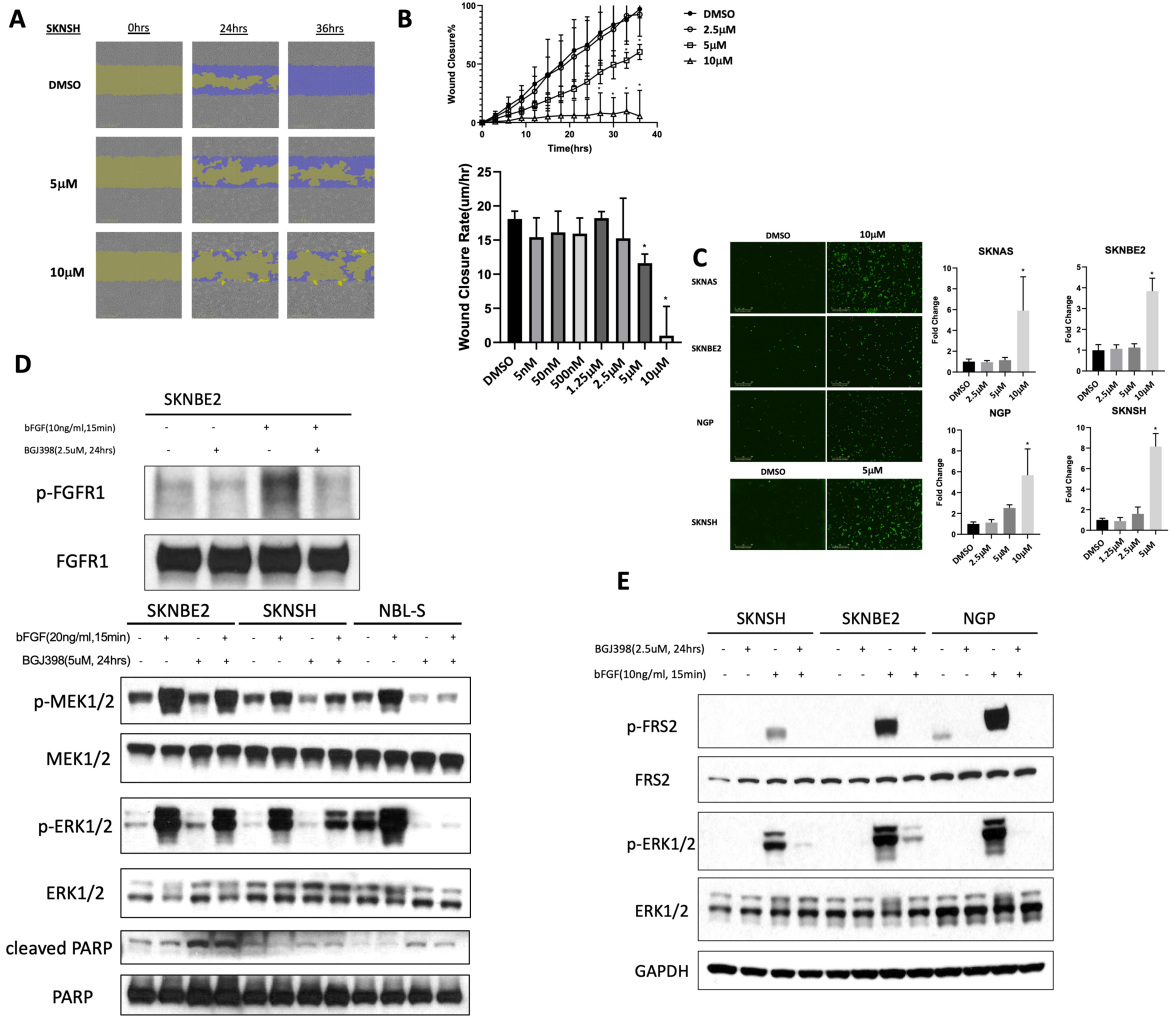
Effect of FGFR inhibitor BGJ398 on neuroblastoma cell migration, cell death, and intracellular signaling. (A) SK-N-SH neuroblastoma cells were treated with increasing concentrations of BGJ398 and uniform scratch wounds were generated. Images from the IncuCyte Zoom^TM^ live-cell imager were obtained following the initial wound and 24h and 36h after treatment with BGJ398, with cells migrating into the scratch wound shown in purple; (B) Wound closure was calculated at regular intervals and plotted over time. Wound closure rates were also calculated and plotted versus concentrations of BGJ398; (C) SK-N-SH neuroblastoma cells were treated with 2.5, 5, or 10 µM BGJ398 for 72 h and were evaluated using a live cell imaging caspase activity assay. Images were obtained and analyzed using the IncuCyte Zoom^TM^, with individual green dots representing cells with caspase activity. Images of untreated cells and of cells treated with 5 or 10 μM BGJ398 for 72 h are shown (left). Average green dot counts per field were normalized to control cells, and fold change was determined by comparing average green object counts in treated cells with average counts in untreated cells (right); (D) (top) SK-N-BE(2) cells were treated with or without BGJ398 (2.5 µM) for 24 h followed by stimulation of bFGF (20ng/ml) 15 min prior to protein extraction. Western blots were performed to determine effects on levels of total and phosphorylated FGFR1. (Bottom) SK-NBE(2), SK-N-SH, and NBL-S neuroblastoma cells were treated with or without BGJ398 (2.5 µM) for 24 h followed by stimulation of bFGF (20ng/ml) 15 min prior to protein extraction. Western blots were performed to determine effects on levels of total and phosphorylated MEK1/2 and ERK1/2 along with PARP and cleaved PARP; (E) SK-N-BE(2), SK-N-SH, and NGP neuroblastoma cells were treated with or without BGJ398 (2.5 µM) for 24 h followed by stimulation of bFGF (20ng/ml) 15min prior to protein extraction. Western blots were performed to determine effects on levels of total and phosphorylated FRS2 and ERK1/2. GAPDH was used as a loading control. FGFR: fibroblast growth factor receptor; PARP: poly-ADP ribose polymerase; FRS: fibroblast growth factor receptor substrate; BGJ398: infigratinib; ERK: extracellular signal-regulated kinase.

BGJ398 at doses over 2.5 μM also inhibited neuroblastoma cell migration [Figure 8A and B; Supplementary Figure 5]. Treatment with 5µM BGJ398 also resulted in a significant increase in apoptosis after 72 h of treatment [Figure 8C]. Finally, downregulation of p-MERK1/2, pERK1/2 and an increase in cleaved PARP were observed in multiple neuroblastoma cell lines after 5 μM BGJ398 treatment for 24 h, suggesting inhibition of FGFR-mediated signaling leading to induction of caspase and PARP cleavage and of apoptosis [Figure 8D].

## DISCUSSION

Prior epidemiological and molecular studies of numerous cancer types have reported a variety of FGFR gene family cytogenetic alterations, including point mutations, chromosomal fusions and rearrangements, chromosomal copy number amplifications, and FGFR gene family member overexpression^[33]^. Alterations in FGFR genes have been detected in approximately 5-10% of unselected patients with cancer. In a recent study of 4,853 adult tumors, Helsten et al. reported that of the 7.1% of FGFR1-4-altered cancers in their cohort of 4853 tumors, 66% of the aberrations were due to copy number alterations, while 26% were single nucleotide variants, and 8% were gene rearrangements or fusions^[34]^, and of 343 patients with an FGFR alteration, 89% of *FGFR1* and 78% of *FGFR4* alterations were amplifications compared with *FGFR2* and *FGFR3* with frequencies of 49% and 30%, respectively. Activating FGFR gene family point mutations and gene fusions have been detected both within and outside the tyrosine kinase domain in adult endometrial, urothelial, and lung cancers^[33]^, and these alterations subsequently drive increased receptor dimerization and ligand-independent activation to promote cancer development^[35]^. The primary effect of these FGFR gene family alterations is to induce constitutive activation of the receptor with subsequent increased activity of downstream signaling pathways, resulting in uncontrolled cell proliferation, migration, and treatment resistance, which are biological hallmarks of cancer.

Both the overall frequency of FGFR gene family member alterations and the distribution of the varied types of FGFR family member alterations vary by cancer type^[6,33,34]^. FGFR alterations detected in solid tumors include *FGFR1* amplifications in non-small-cell lung cancer (in 20% of patients), *FGFR1/2* amplifications in breast cancer (7-23%), *FGFR3* mutations (10%-60%) or *FGFR3* fusions (6%) in urothelial carcinoma, *FGFR2* fusions in intrahepatic cholangiocarcinoma (10%-20%), *FGFR2* mutations in endometrial uterine cancer (12%) and *FGFR2* amplifications in gastric cancer (5%-10%), which are representative of the spectrum of FGFR abnormalities in adult cancers^[6,36]^. FGFR gene fusions have also been reported in a wide range of adult cancers, including lung, breast, pancreatic, prostate, and thyroid cancers, among others, but these tumors are rarely found in children^[33,34,37]^.

Prior studies of pediatric tumor samples have determined that FGFR genomic aberrations are present but are both rare and recurrent, ranging from 0.3% to 5% in published cohorts. Genomic FGFR alterations are enriched in specific subtypes of pediatric gliomas^[34]^ and sarcomas^[38]^ that frequently have poor outcomes, but the prevalence and distribution of these alterations are significantly different from those seen in adult cancers. Oncogenic FGFR signaling has been found in 4% of pediatric brain tumors and up to 11% in pediatric low-grade glioma patients^[39]^, and of all FGFR-altered pediatric gliomas, 42% contain internal tandem duplications in *FGFR1*^[40]^. In rhabdomyosarcoma tumors without PAX3-FOXO1 gene fusions, *FGFR4* mutations are found in 10% of cases, while nearly all tumors have elevated *FGFR4* expression^[8,41]^. In a recent study of large cohorts of pediatric solid tumors comprising 1,395 patients^[7]^, oncogenic FGFR alterations were identified in 41 tumors, including 11 rhabdomyosarcomas, nine low-grade gliomas, and 17 other tumor types. One neuroblastoma tumor had an *FGFR2* missense mutation. These FGFR family alterations included gain-of-function sequence variants (*n* = 19), amplifications (*n* = 10), oncogenic fusions [FGFR3:TACC3 (*n* = 3), FGFR1:TACC1 (*n* = 1), FGFR1:EBF2 (*n* = 1), FGFR1:CLIP2 (*n* = 1), and FGFR2:CTNNA3 (*n* = 1)], pathogenic-leaning variants of uncertain significance (*n* = 4), and amplification in combination with a pathogenic-leaning variant of uncertain significance (*n* = 1), identifying a population of children with cancer who may be good candidates for FGFR inhibitor treatment. In this study, we sought to further characterize the prevalence and distribution of FGFR gene alterations in pediatric solid tumors and identify and characterize differences between pediatric and adult cancers. Currently, identifying patients most likely to benefit from FGFR inhibition depends on detecting activating FGFR mutations, gene fusions, or gene amplification events. While identification of rare FGFR gene alterations is challenging due to the small numbers of patients in most cohorts of pediatric patients in available databases, the full spectrum of these FGFR gene family alterations in pediatric cancers is emerging as comprehensive DNA sequencing is more frequently employed for both tumor and patient evaluation, and the expanding use of RNA-sequencing has identified more frequent occurrences of tumors that overexpress FGFR family members in the absence of any genomic alteration. However, the associations of FGFR gene family member overexpression with FGFR oncogenic activity remain unclear.

FGFR gene family alterations promote tumorigenesis through aberrant FGFR signaling in a variety of ways. FGFR gene family amplifications have been shown to drive FGFR overexpression and increased activity of downstream signaling pathways, while FGFR gene fusions and chromosomal rearrangements frequently enhance receptor dimerization or independently induce aberrant downstream intracellular signaling. FGFR gene family mutations in the extracellular or transmembrane domains can also activate intracellular signaling by enhancing the affinity for ligand binding or inducing ligand-independent signaling, while mutations in the kinase domain lead to constitutive activation and disruption of inhibitory mechanisms^[42]^.

Expression of FGF ligands and expression and oncogenic activity of FGFR family members have been associated with resistance to numerous standard chemotherapy agents utilized for treatment of both adult and pediatric cancers, including cisplatin, doxorubicin, and etoposide^[43-53]^, and FGFR gene family overexpression in cancer cells has also been associated with reduced responses to targeted kinase inhibitors and to endocrine therapy in adult cancer patients^[54-60]^. However, while the increased FGF ligand and FGFR family member expression observed in many cancers is often associated with resistance to anti-cancer therapy, the exact mechanisms of this resistance are not well understood.

Activation of FGFR family members via either ligand binding or genetic alteration leads to the activation of a number of intracellular signaling pathways that may affect the responses of tumors to treatment. FGFR-mediated signaling regulates critical cellular processes such as proliferation, differentiation, migration, and apoptosis, which contribute to embryonic development as well as to the maintenance of homeostasis in adult tissues^[61-63]^. FGFR activation by ligand binding leads to receptor dimerization and recruitment and activation of adaptor proteins, including the fibroblast growth factor receptor substrate 2 (FRS2α) and GRB2^[62]^. Activated GRB2 can then recruit SOS1 or GAB1, leading to activation and signaling through the RAS/MAPK and PI3K/AKT/mTOR pathways, respectively^[62,64]^. FGFR activation also induces activation of the JAK/STAT and PLCγ/PKC pathways, among many others^[62]^. FGFR signaling promotes metastasis of the pediatric solid tumor rhabdomyosarcoma^[65]^, and signaling pathways activated by FGFRs can also inhibit apoptosis, thereby reducing the efficacy of some anti-cancer agents, while FGFR-mediated signaling also initiates angiogenesis and epithelial-to-mesenchymal (EMT) pathways that have been shown to be associated with treatment resistance^[5]^. These signaling pathways represent opportunities for synergistic drug combinations. It has been observed in a number of cancer genome sequencing studies that mutations in major oncogenic drivers are often mutually exclusive, especially if the oncogenes participate in the same signal transduction pathways^[66]^. The mechanism underlying this mutual exclusivity remains to be elucidated, but a plausible explanation is that the co-activation of multiple oncogenes may lead to cell senescence^[67]^. In our study, we also observed a similar pattern [Figure 1]. For instance, alterations in *FGFR1-4*, *IDH1/2*, *KRAS*, *ALK*, *ROS1*, *NTRKs*, and *BRAF* genes are all largely mutually exclusive. This suggests that once the main driving mutation is identified, single-agent targeted therapy (combined with standard-of-care chemotherapy if necessary) is likely the preferred option. In rare cases when two or more oncogenic events are simultaneously present, combination therapy may be considered, but the exact dosing regimen, including the dosages and sequencing, may need to be developed for each individual patient.

Treatment resistance associated with FGFR gene family member alterations is the result of activation of downstream intracellular signaling pathways, including the RAS/MAPK pathway, the most common pathway associated with FGFR-mediated treatment resistance^[57,68]^, and the PI3K/AKT/mTOR pathway, which has previously been identified as playing a key role in FGFR-dependent tumor progression and treatment resistance^[69-71]^. Activation of the RAS/MAPK pathway can further induce stabilization of Twist and a switch from E-cadherin to N-cadherin expression, along with upregulation of the expression of other mesenchymal markers and transcription factors driving the EMT process. Activation of the JAK/STAT pathway has also been found to be correlated with resistance to chemotherapy and to inhibitors of the RAS/MAPK pathway in a variety of adult cancers^[72]^. Inhibition of apoptosis driven by FGFR gene family member alterations is typically the result of increased expression of Bcl-2 and Bcl-xL or through stabilization and increased expression of MDM2 along with inhibition of Bad, Bax, and other activators of apoptosis. FGFR alterations can also drive enhanced angiogenesis via increased expression of VEGF and HGF. Furthermore, the FGFR4-R388 mutation can enhance interactions with matrix metalloproteinases and other proteins that drive tumor invasion and metastasis^[5]^. FGFR inhibition in patients with cancer is, therefore, likely to be effective via effects on multiple intracellular signaling pathways. However, this approach may also lead to the development of resistance through the acquisition of specific mutations or via indirect effects on and compensation from alternative intracellular signaling networks.

Multiple FGFR-directed therapies are in various clinical stages, including small molecule receptor tyrosine kinase inhibitors (TKIs), monoclonal antibodies, and FGF ligand traps^[73]^. The first-generation TKIs were typically non-covalent, pan-FGFR inhibitors, such as pemigatinib, erdafitinib (JNJ-42756493), and BGJ398^[74,75]^. Development of the second generation of FGFR TKIs is ongoing and aimed at overcoming resistance and improving binding kinetics. To potentially address resistance mediated by point mutations, especially gatekeeper mutations, irreversible FGFR inhibitors are being developed. One example is KIN-3248, an irreversible FGFR1-4 inhibitor designed to block both primary oncogenic and secondary kinase domain resistance FGFR alterations. However, none of these newer FGFR inhibitors have been approved. For resistance caused by activation of alternative signaling pathways, a broad-spectrum, multi-TKI inhibitor may help, but it depends on the targeting spectrum of these compounds and their safety profile associated with their promiscuity. Futibatinib (TAS-120) exemplifies this newer generation with covalent binding within the P-loop of FGFR^[76]^. Clinical benefit from these agents is in part limited by hyperphosphatemia owing to FGFR1 inhibition, as well as the emergence of resistance mutations in FGFR genes, activation of bypass signaling pathways, concurrent *TP53* alterations, and possibly, epithelial–mesenchymal transition-related isoform switching. Most recently, FGFR subtype-specific inhibitors have entered clinical development. These inhibitors typically prioritize inhibition of FGFR2 or FGFR3 while sparing FGFR1, with the goal of reducing FGFR1-mediated side effects widely observed in pan-FGFR TKI trials^[77,78]^. Besides TKIs, monoclonal antibodies^[79]^, antibody-drug conjugates (ADCs)^[80]^, ligand traps^[81]^, and protein degraders^[82]^ are also being investigated. Among these, some TKIs have demonstrated clinical benefits in FGFR-driven intrahepatic cholangiocarcinoma and urothelial cancer^[83]^. In spite of the difference in the type of alterations and the prevalence of genetic alterations between adult and pediatric cancers, the mechanism of FGFR activation between adult and pediatric cancers is similar, and patient age did not have an effect on the mode of activation or signaling pathway for FGFR genetic alterations^[78]^. Therefore, the FGFR-targeting agents for adult tumors should be active in FGFR-driven pediatric cancers if used appropriately.

Acquired resistance to direct FGFR inhibition is a key limiting factor to the long-term efficacy of FGFR-targeted therapies. Treatment-selected or -induced mutations are a common resistance mechanism for most small-molecule TKIs. The extent of clinical benefit from FGFR inhibitors can be compromised by several types of resistance mutation, including gatekeeper, molecular-brake and DFG-latch mutations, which limit the ability of FGFR inhibitors to access drug-binding pockets and thus enable the mutant FGFR proteins to remain active. Mutations to FGFR “gatekeeper” residues, such as *FGFR1* V561M and *FGFR2* V565I, lead to steric hindrance within the kinase binding pocket, precluding the entry and binding of FGFR inhibitors^[6]^. Besides gatekeeper mutations, additional point mutations such as *FGFR3* V555L, V555M, N540K, L608V, and K650E have been detected, although the exact biological significance of these alterations is yet to be elucidated^[6,84]^. Furthermore, acquired resistance can often involve non-FGFR-mediated signaling activity to bypass FGFR-mediated signaling. In some cases, MET, PI3K/AKT, or EGFR pathways are activated to overcome TKI-mediated inhibition of FGFR signaling. Combination treatment, either simultaneous or sequential, may help delay or mitigate resistance due to these “bypass” pathways, and further studies are clearly indicated to determine the associations of FGFR expression and signaling pathway activity with pediatric cancer cell responses to BGJ398 and other FGFR inhibitors.

We have shown that FGFR genetic alterations are rare events in pediatric solid tumors. Based on clinical experience in adult cancers, it has become clear that aberrant FGFR-mediated signaling is predictive of response to FGFR inhibitors in urothelial cancer (mainly driven by *FGFR3* mutations) and cholangiocarcinoma (mainly driven by *FGFR2* fusions). Therefore, it will be equally important to molecularly define pediatric patients for FGFR inhibitor therapy^[85]^. While the results of our studies are clearly limited by both the relatively small numbers of pediatric cancer patients and available pediatric tumor datasets, along with the rarity of mutations and other aberrations in FGFR family member genes, the continued expansion of both the numbers and sizes of available databases will allow for more detailed exploration of the role of FGFR alterations in pediatric solid tumors. The detection of somatic FGFR alterations can be potentially achieved by fluorescence in situ hybridization (FISH)^[86]^, immunohistochemistry (IHC)^[87]^, RT-PCR^[87]^ and next-generation sequencing (NGS)^[88]^, which are all currently in use for pediatric tumor evaluation. Furthermore, while we have demonstrated the efficacy of a single pan-FGFR inhibitor (BGJ398) against pediatric solid tumor cells and associations with FGFR family member expression, a full understanding of the roles of different FGFR family members will require testing of more specific inhibitors. Further studies are ongoing to more fully characterize the mechanisms by which altered intracellular signaling by FGFR inhibition in pediatric solid tumor cells leads to effects of cell migration and viability. In summary, targeting the FGFR signaling pathway has the potential in treating FGFR-driven pediatric solid tumors, and more proactive clinical development efforts, including innovative diagnostic approaches and age-adjusted regimens, are needed to realize the potential value of these targeted agents.

## DECLARATIONS

### Availability of data and materials

Publicly available datasets were analyzed in this study. The data can be found at the R2 Genomics Analysis and Visualization Platform (http://r2.amc.nl), cBioPortal databases (http://www.cbioportal.org), and the Foundation Medicine database (https://corpsite.foundationmedicine.com/insights-and-trials).

### Copyright

© The Author(s) 2025.
