# Supplementary material for: Fibroblast growth factor receptor alterations and resistance mechanisms in the treatment of pediatric solid tumors
Source: Cancer Drug Resist. 2025 Jun 6;8:28. doi: 10.20517/cdr.2024.208 (PMC12159601; doi:10.20517/cdr.2024.208)
Supplement: Supplementary file 1 [file cdr-8-28-SupplementaryMaterials.pdf]

Supplementary Materials

Fibroblast growth factor receptor alterations and resistance mechanisms in the treatment of pediatric solid tumors

Ivan Li[1,#], Yuchen Huo[2,#], Ting Yang[3], Howard Gunawan[2], Ludmil B. Alexandrov[3], Peter E. Zage[2,4]

[1]Tufts University, Medford, MA 02155, USA.

[2]Department of Pediatrics, Division of Hematology-Oncology, University of California San Diego, La Jolla, CA 92093, USA.

[3]Departments of Cellular and Molecular Medicine and Bioengineering, University of California San Diego, La Jolla, CA 92093, USA.

[4]Peckham Center for Cancer and Blood Disorders, Rady Children's Hospital, San Diego, CA 92123, USA.

[#]They contributed equally.

Correspondence to: Dr. Peter Zage, University of California San Diego School of Medicine; Moores Cancer Center, Room 5311; 3855 Health Sciences Dr, MC 0815; La Jolla, CA 92093-0815; Email: pzage@health.ucsd.edu

ORCID: Peter Zage (0000-0003-1837-2315)

Figures

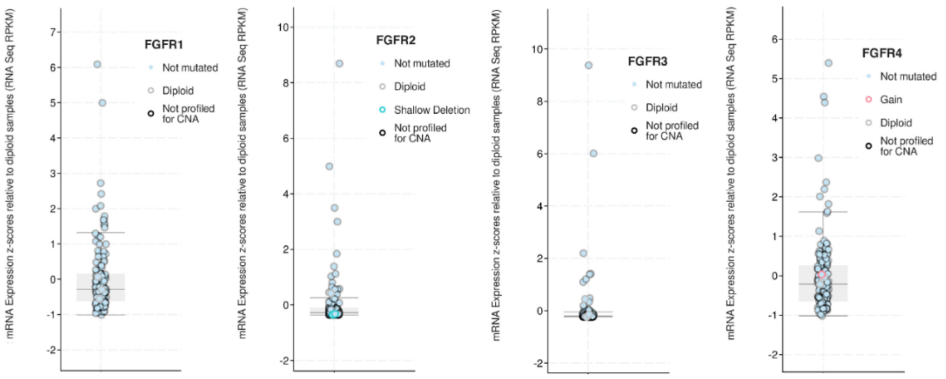

Figure S1. Overexpression of FGFRs in neuroblastoma. Queries and resulting figures were generated in cBioPortal (http://www.cbioportal.org) by searching “neuroblastoma” and selecting the TARGET database[92] and querying by *FGFR1*, *FGFR2*, *FGFR3*, and *FGFR4*, with the resulting expression for each shown.

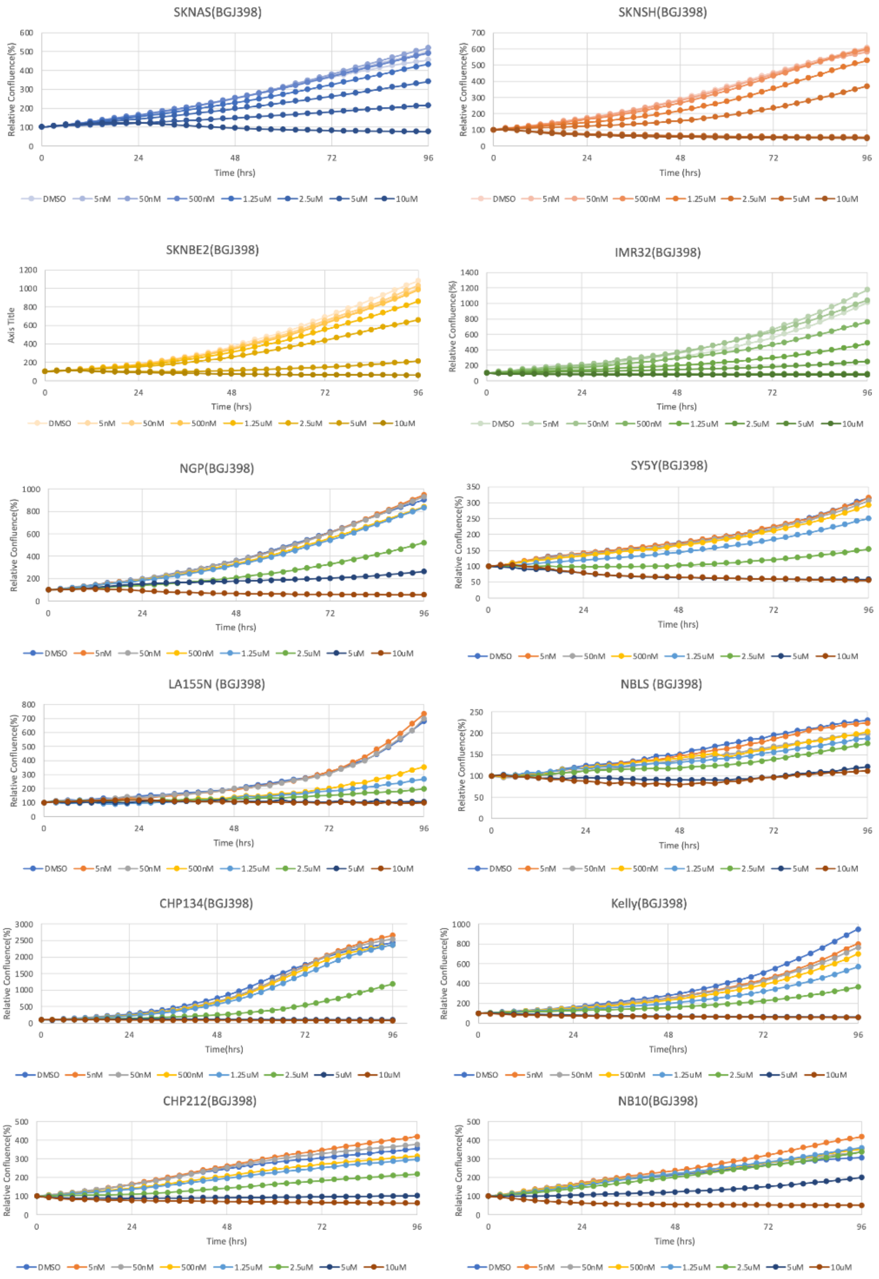

Figure S2. Effect of FGFR inhibitor BGJ398 on neuroblastoma cell confluence. Neuroblastoma cell lines (SK-N-AS, SK-N-SH, SK-N-BE(2), IMR-32, NGP, SH-SY5Y, LA155N, NBL-S, CHP134, Kelly, CHP212 and NB-10) were treated with increasing concentrations of BGJ398. Cell confluence was assessed by continuous

live cell imaging over 96h of incubation, as previously published. Time- and dose-response curves are shown.

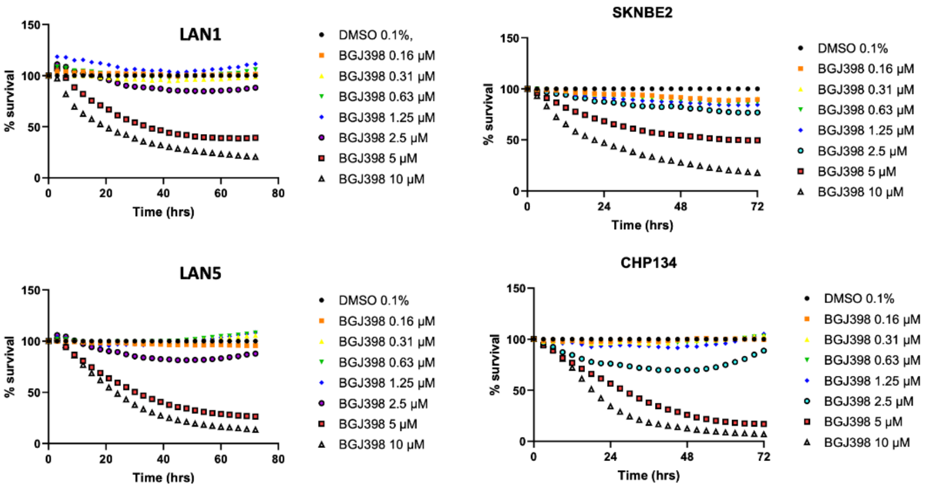

Figure S3. Effect of FGFR inhibitor BGJ398 on neuroblastoma cell survival. Neuroblastoma cell lines [SK-N-BE(2), CHP134, LAN1, and LAN5] were treated with increasing concentrations of BGJ398. Cell confluence (% survival) was assessed by continuous live cell imaging after 72 h of incubation, with time- and dose-response curves shown.

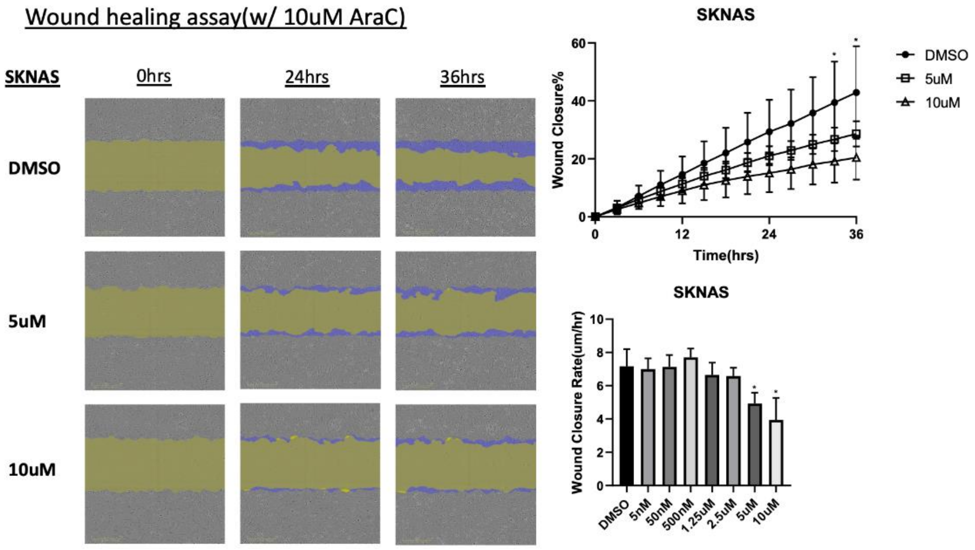

Figure S4. Effect of FGFR inhibitor BGJ398 on pediatric sarcoma cell survival. (A) Ewing sarcoma (A673, TC32, TC71) and (B) rhabdomyosarcoma (Rh30, Rh41, RD) cell lines were treated with increasing concentrations of BGJ398. Cell confluence was

assessed by continuous live cell imaging after 72 h of incubation, with time- and dose-response curves shown. (C) Ewing sarcoma and rhabdomyosarcoma cell lines were treated with increasing concentrations of BGJ398 for 72 h. Mean average values for a reduction in confluence (% inhibition) from triplicate experiments were then plotted against BGJ398 dose levels. (B) IC50 values were calculated using curve-fit equations for each tested cell line listed from lowest to highest IC50 values for each cell type.

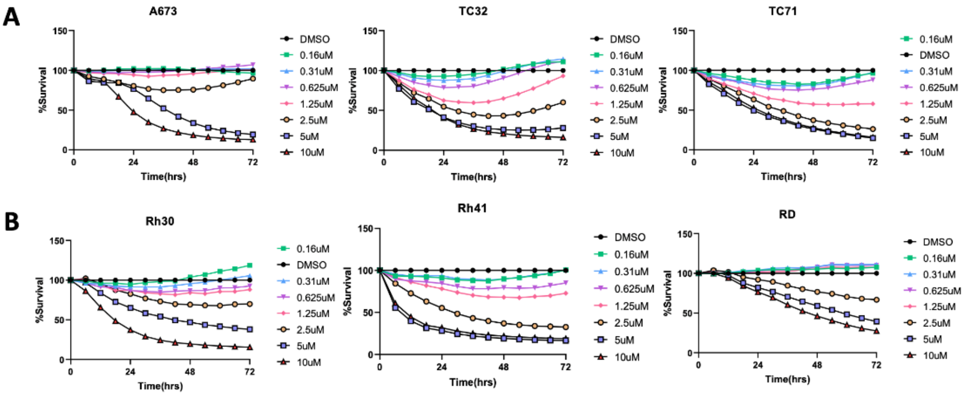

C

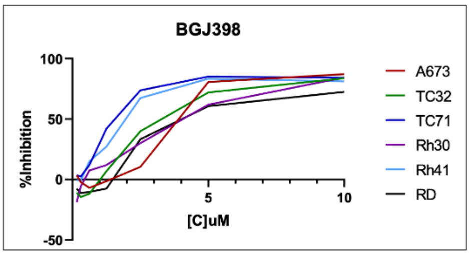

Cell lines	IC50 (uM)
TC71	1.54
TC32	3.20
A673	3.56
Rh41	1.95
RD	3.81
Rh41	3.90

Figure S5. Effect of FGFR inhibitor BGJ398 on neuroblastoma cell migration. (Left) SK-N-AS neuroblastoma cells were treated with increasing concentrations of BGJ398 and uniform scratch wounds were generated. Images were obtained from the IncuCyte Zoom^{TM} following the initial wound and 24h and 36h after treatment with BGJ398, with cells migrating into the scratch wound shown in purple. (Top right) The percentage of wound closure was calculated at regular intervals and plotted over time. (Bottom right) Wound closure rates were also calculated and plotted compared to concentrations of BGJ398.
